# Sexual harassment in academe is underreported, especially by students in the life and physical sciences

**DOI:** 10.1371/journal.pone.0230312

**Published:** 2020-03-10

**Authors:** Stephen J. Aguilar, Clare Baek

**Affiliations:** Rossier School of Education, University of Southern California, Los Angeles, California, United States of America; University of Westminster, UNITED KINGDOM

## Abstract

What factors predict the underreporting of sexual harassment in academe? We used logistic regression and sentiment analysis to examine 2,343 reports of sexual harassment involving members of university communities. Results indicate students were 1.6 times likely to not report their experiences when compared to faculty. Respondents in the life and physical sciences were 1.7 times more likely to not report their experiences when compared to respondents in other disciplines. Men represented 90% of the reported perpetrators of sexual harassment. Analysis of respondents’ written accounts show variation of overall sentiment based on discipline, student type, and the type of institution attended, particularly with regard to mental health. Our results suggest that institutional and departmental barriers driven by power asymmetries play a large role in the underreporting sexual harassment among students—especially those in STEM disciplines.

## Introduction

Sexual harassment in the workplace is pervasive and disproportionally experienced by women. The majority of sexual harassment reports made to the Equal Employment Opportunities Commission (EEOC), for example, have been initiated by women [1] and 60% to 75% of women report having experienced “unwanted sexual attention or sexual coercion” in the workplace [2]. Recent studies have shown similar patterns for college students—50% of female students experienced some kind of sexual harassment during their college years, and women of color have been shown to experience especially high rates of harassment [3–4]. In the academy, the majority of reported sexual harassment involves unwelcome physical contact (e.g., groping, sexual assault, and domestic abuse-like behaviors) by repeat offenders [5]. Women in the academy are also more likely to be the targets of inappropriate sexual comments from supervisors during fieldwork [6], and are often targeted when they are trainees [7].

Those who experience sexual harassment in post-secondary settings suffer mental, psychological, physical, academic, and work-related consequences—including lower GPA for students and, in the case of faculty, potentially leaving academe altogether [8–11]. The negative effects of sexual harassment, moreover, can affect an entire scholarly community; recent work has shown that an environment where sexual harassment is pervasive can lead to faculty burnout among women, even if they do not experience sexual harassment directly [12].

Yet, despite the pervasiveness of sexual harassment in academic settings, evidence indicates that reporting rates in academe are generally low, potentially due to fear of retaliation—especially when the perpetrator is a prominent scientist [13]. Such examples signal the role of power imbalances between perpetrator and those whom they victimize. Undergraduate students, for example, report sexual harassment less often when the perpetrator is a faculty member vs. a fellow student [14], and also report sexual harassment less overall compared to their graduate student counterparts [3].

Reporting rates are also influenced by a process that often compels university employees to report student sexual assault disclosures, even when students wish to maintain confidentiality and despite limited evidence that compulsory reporting is beneficial to the students themselves [15]. Such policies may explain recent evidence that shows that students who are knowledgeable about the Title IX reporting process still choose to not report incidents of sexual harassment in hypothetical scenarios [16]. Compulsory reporting, moreover, has been argued to be in conflict with professional ethics codes and practices, such as those of the American Psychological Association [17].

The attitudes of university leadership, a university’s prior response to sexual harassment reports, and prior outcomes for those who reported sexual harassment also play a prominent role in reporting behaviors [18–20]. An organizational climate that exhibits tolerance of sexual harassment, for example, may have leaders with dismissive attitudes that undermine formal (or informal) reporting processes. This perceived dismissiveness has been shown to deter victims and whistleblowers from reporting sexual harassment [21]. In contrast, evidence suggests that the presence of a clear no-tolerance policy increases reporting [19].

Given the high rate of sexual harassment in the academy, there is a renewed and vigorous call for action to address—and prevent—it [22]. Faculty accused of sexually harassing their graduate students have been placed on leave pending investigation [23–25]. Those found guilty are sometimes stripped of academic honors [22, 26], or recommended for termination [23–25]. Funding agencies have also taken steps to address sexual harassment; the National Science Foundation, for example, has established new reporting requirements that will enable it to track sexual harassment committed by its grantees [27]. Despite these positive steps, a report by the National Academies of Science, Engineering, and Medicine found sexual harassment in the academy to be pervasive, and concluded that organizational climate is a potent predictor of it [28].

Addressing and preventing sexual harassment in the academy requires a better understanding of what factors contribute to reporting behaviors so that sound policy can be written to address it. The present study responds to calls for more research in this area (e.g., [17]), by examining predictors of non-reporting behaviors and providing a descriptive analysis of the sentiment used in narrative statements describing sexual harassment experiences gathered through a crowed-sourced survey of sexual harassment in higher education.

### Sentiment analysis

Sentiment analysis uses natural language processing to analyze writing that contains opinions, emotions, and attitudes towards topics of interest like products, events, and organizations. The process quantifies the degree of positive, negative, or neutral sentiment contained within the text, and can be applied to varying text segment lengths [29–30]. Document-level analysis, for example, classifies a whole document as generally positive, negative or neutral, whereas sentence-level analysis does the same for a sentence [29]. This process helps to reveal the intensity of the writers’ opinions on a topic of interest (e.g., [30–33]). Chen, Zhu, Kifer, and Lee [34], for example, used sentiment analysis to compare the opinions of Republican and Democratic senators with respect to a variety of topics, focusing on similarities and dissimilarities between the two groups’ opinions. Other work has found differences between men and women when it comes to the emotional content of work-place email [35]. Sentiment analysis has also been used to investigate the mental health status reflected through written language (e.g., [36]), and has been used with geographic information system data to identify communities in need of mental health services in the wake of natural disasters [37].

### Current study

We contribute to studies on sexual harassment (e.g., [19, 16, 38–41]) by focusing on reporting behaviors in the academy, and the sentiment present when describing harassment. Specifically, we analyzed unique reports of sexual harassment that occurred in higher education settings across diverse disciplines and institution types. These reports were collected through a crowdsourced survey, where the respondents shared the accounts of perceived sexual harassment through their answers to open-ended questions. We focus on characteristics that predict the reporting behaviors in order to identify potential trends across academic disciplines, and our analysis answers the following research questions:

**RQ1:** Which respondent, perpetrator, and/or institutional characteristics to predict the non-reporting behavior of sexual harassment?

**RQ2:** What is the sentiment of respondents’ answers to open-ended questions about sexual harassment experiences?

## Method

### Survey instrument

This study was approved in accordance with the University of Southern California’s IRB guidelines (UP-18-00179). The data used for our analysis was gathered through an anonymous survey written and disseminated by Dr. Karen Kelsky—a consultant who focuses on helping individuals navigate the Ph.D. job market and advocate for marginalized groups within the academy. Her stated goal for the survey was “…for the academy as a whole to begin to grasp the true scope and scale of [sexual harassment] in academic settings,” [42]. The survey was launched in November 2017 on Google Forms, and shared through social media posts. The survey did not have a formal close date; responses were made publicly available and were downloaded February 2018. The crowdsourced survey captured responses from many individuals (n = 2,343) from a variety of institutions all around the country, and beyond. The survey captures direct and second-hand experience of sexual harassment in the academy at various points in time in a person’s academic career (e.g., undergraduate, graduate, faculty).

#### Demographic items

Respondents answered demographic questions that included their status during an incident of sexual harassment (e.g., graduate student); the status of the perpetrators (e.g., tenured professor); the gender of the perpetrator; the type of institution where the harassment occurred (e.g., “R1 institution”); and the respondent’s field of study.

#### Reporting

Respondents answered one item that asked whether they reported the incident to their institution, and the institution’s response if the incident was reported.

#### Open-ended questions

Respondents also answered four open-ended questions that focused on: 1) what happened, and when it happened—what we term the “harassment narrative”; 2) the impact the harassment had on their career; 3) the impact the harassment had on their mental health; and 4) the impact the harassment had on their choices or life trajectory.

#### Sample

The majority of respondents were from U.S. based institutions. The data captures instances of sexual harassment that may not have been reported to the institution where it occurred, as well as respondents’ reflections on the impact the incident had on various dimensions of their lives. The data reflect 2,343 unique respondents and 9,372 harassment, mental health, career impact, and life trajectory narratives (combined).

#### Respondents

The survey did not formally ask respondents to indicate their sex or gender. Many of the respondents, however, used feminine pronouns (e.g., she/her) or otherwise described those who experienced sexual harassment as “women” or “female” in their description of sexual harassment. These responses were coded for the presence of such pronouns in the narrative statement. A second rater coded a sub-sample (n = 434) to calculate agreement; Cohen's κ was run to determine if there was agreement between the two raters’ judgments. There was moderate agreement (73%), κ = .570, p < .001. Given thus, we infer that approximately 43% of respondents reported that women were the targets of the sexual harassment experience. The majority of respondents (70%) were students at various stages of their academic careers during the alleged harassment, (e.g., undergraduate or graduate); 20% were faculty, and 10% were staff (see Table 1 for further breakdown). Respondents came from different fields, including the humanities (47%), the social sciences (26%), the physical sciences (16.9%), and professional schools (7%) (see Table 2).

**Table 1:** Respondent demographic characteristics.

	N	Percentage
**Faculty**	**451**	**19.8%**
Assistant Professor	266	11.7%
Associate Professor	35	1.5%
Full Professor	25	1.1%
Adjunct Professor	41	1.8%
Lecturer	51	2.2%
Faculty*	33	1.5%
**Student**	**1,602**	**70.3%**
High School Student	8	0.4%
Undergraduate Student	352	15.4%
Masters Student	127	5.6%
PhD Student	438	19.2%
Graduate Student*	565	24.8%
Postdoc	59	2.6%
Student*	53	2.3%
**Misc.**	**227**	**9.9%**
Staff	64	2.8%
Multiple	122	5.4%
Other	41	1.8%
Not reported	54	2.3%

Note

* = only generic term used, further details not specified. Demographic breakdown of participants of MeTooPh.D. Survey Respondents (n = 2,343)

**Table 2:** Discipline of respondents.

	N	Percentage
Humanities	925	47.0%
Social Sciences	517	26.3%
Physical Sciences	332	16.9%
Engineering	32	1.6%
Professional	139	7.1%
Staff	24	1.2%
Not reported	325	13.8%

#### Reported perpetrators

The vast majority of reported perpetrators were men (92%), with 73% of them also identified as faculty. Faculty perpetrators had various supervisory roles (e.g., advisors), and 35% of perpetrators were identified as being tenured (see Table 3 for more detailed breakdown of perpetrator statistics).

**Table 3:** Perpetrator demographic characteristics.

	N	Percentage
**Gender**		
Male	2,114	91.79%
Female	117	5.08%
Mixed Group	43	1.87%
Other	29	1.26%
**Rank/Role**		
Assistant Professor	107	4.7%
Associate Professor	108	4.7%
Full Professor	576	25.2%
Graduate Student	165	7.2%
Postdoc	23	1.0%
PhD Student	92	4.0%
Faculty*	874	38.3%
Other	338	14.8%
**Supervisory Positions**		
Advisor/Mentor	209	9.2%
Chair/Department Head	209	9.2%
Dean	22	1.0%
Principal Investigator	25	1.1%
Tenured	742	32.5%
Endowed Chair/"Famous"	118	5.2%
Other supervisory role	247	10.8%
Not reported	51	2.2%

Note

* = only generic title written, further details not specified. For gender, “other” refers to responses that are unclear or not reported

#### Location

Harassment took place across various institution types: 25% of respondents reported experiencing sexual harassment at R-1 research institutions, 40% at Ivy or “Elite” institutions, and 33% at other intuitions (see Table 4 in for a full list).

**Table 4:** Reported institution type.

	N	Percentage
Elite Institution/Ivy League	577	25.36%
More than one institution	120	5.27%
Other R1	928	40.79%
Other Research Agency	52	2.29%
Other Type of School	167	7.34%
R2	151	6.64%
Regional Teaching College	76	3.34%
Small Liberal Arts College	203	8.92%
[redacted]	1	0.04%
Not reported	68	2.90%

The two largest categories (“Elite Institution/Ivy League” and “Other R1”) were maintained for analysis. All other categories were combined into “Other.” Note: “redacted” category from original survey results; the research team did not redact any data.

### Data preparation

#### Sentiment scores generation for open-ended responses

Text data (RQ2) were processed in R using the Text Mining (TM) package [43–45]. This process enables quantitative analysis of text, and also removes common stop word (e.g., the, it, a) to facilitate analysis. We then used the “SentimentAnalysis” R package [46] to conduct sentiment analysis on each of open-ended responses of the data set; this process generates a sentiment score between -1 (negative) and 1 (positive) for each of the narratives using existing dictionaries. Sentiment variables were generated for each of the passages below.

Harassment Narrative. Respondents to the survey were asked: “What Happened and When?” The mean word length of responses was 57 (SD = 70).

Impact on Career Narrative. Respondents to the survey were asked: “The Impact of the Harassment on Your Career.” The mean word length of responses was 11 (SD = 15)

Impact on Mental Health Narrative. Respondents to the survey were asked: “The Impact of the Harassment on Your Mental Health.” The mean word length of responses was 11 (SD = 18)

Impact on Career Trajectory Narrative. Respondents to the survey were asked: “The Impact of the Harassment on Your Life Choices/Trajectory.” The mean word length of responses was 10 (SD = 17).

#### Manually coded variables

We manually coded discipline and institutional response variables since many of them (e.g., the respondent’s status within the university) were written as free responses in the survey, instead of pre-determined categories that respondents could select from. These variables were manually coded using the process below.

Discipline. Disciplines reported by respondents were aggregated into 6 different categories based on the National Science Foundation’s categories of earned doctorates [47] (see Table 5.)

**Table 5:** Disciplines reported by respondents, assigned category by research team, and coded value for analysis.

Coded Value	Assigned category	Disciplines reported
1	Humanities	Applied Linguistics, Archaeology, Art, Art History, Arts, Classics, Creative Writing, Dance, English, Film Studies, Fine Arts, Foreign Language, History, Liberal Arts, Linguistics, Literature, Music, Performing Arts, Philosophy, Religion, Theatre, Theology, Visual Arts
2	Social Science	Anthropology, Architecture, Communication, Economics, Geography, Information Science, Political Science, Psychology, Sociology, Women Studies
3	Life & Physical Science	Astronomy, Biology, Chemistry, Computer Science, Earth Science, Environmental Studies, Geology, Geoscience, Mathematics, Neuroscience, Oceanography, Paleontology, Physics, Science, STEM
4	Engineering	Chemical Engineering, Mechanical Engineering
5	Professional	Accounting, Business, Criminal Justice, Criminology, Education, Law, Marketing, Medical School, Pharmacology, Public Health,
6	Staff	Administration, Community Service, Development, IT, Library, Student Affairs

Institutional response. The survey question read: “Institutional Responses to the Harassment (If Any).” Responses were coded into six categories based on existing literature, e.g., retaliation, taking actions to redress the situation, and not taking any action (e.g., [18, 48]). A second rater coded a random-sample of 10% of the responses to calculate agreement; Cohen's κ was run to determine if there was agreement between the two raters’ judgments. There was moderate agreement (64%), κ = .512, p < .001. These coded categories were (see Table 6 for more examples):

- **None:** Respondents did not report a formal response by the institution, e.g., “None,” “Nothing has been done,” and “No formal response.”
- **Did not report:** Respondents did not report the incident, e.g., “Never reported it,” “I didn’t say anything,” and “I never told anyone.”
- **Action taken:** Respondents reported an institutional response after the incident was reported, e.g., “Title IX investigation,” “He was fired,” “He was removed.”
- **Unclear, Not sure:** Respondents’ indicated an unclear institutional response, e.g., “Unclear,” “Unknown,” “I think there was an investigation but I never heard any results.”
- **Retaliation (against the respondent):**. Respondents reported retaliation against them for reporting the incident, e.g., “I was silenced and punished,” “I was fired,” “Retaliation.”
- **Other:** Responses that do not fall into the above categories, e.g., “There were conflicting responses from faculty,” “bathroom renovation over a year after the fact.”

**Table 6:** Institutional response categories.

Coded Value	Assigned category	Example Responses
1	None	None; There were no consequences; Nothing was done to him; Disbelief; They said they couldn’t do anything about it; Nothing has been done; There was not enough evidence to pursue and it was dropped; They ignored it; They said I couldn’t be helped; Nothing; They said to ignore it; No formal response; No observed differences in authority after reporting of incidents
2	Did not report	No reporting system for faculty/grad student interactions at the time; Discouraged from reporting due to potential damage to my own career; Never reported it; I didn’t say anything; I did not report it; I did not report because I feared direct retribution; I never told anyone about this; Warned that it would get ugly if I filed a complaint so I didn’t; Not applicable as happened at conferences
3	Action taken	Title IX investigation; He was fired from his tenure position; There is supposedly “a file” on the incident somewhere; He was disciplined; This was enough to force the perpetrator to “retire” with immediate effect; He was removed from my tenure committee and was told to have no contact with me; Forced resignation
4	Unclear, Not sure	I heard stories about “talkings to” that he received but I don’t know; Dean collected information anonymously from myself and other victims; My hope is that this curbed his behavior significantly but I can’t know for sure; I think he was rebuked; No information whether action was taken; Unclear; Unknown; I think there was an investigation but I never heard of any results; They spoke with him beyond that none to my knowledge
5	Retaliation (against the respondent)	I was silenced and punished; Poorly handled title ix investigation where i was neglected by the staff; I was fired; Retaliation; termination after positive review and seizure of external research funds that I won; They began to exclude me
6	Other	Negotiated agreement between me and the harasser; A conversation with the harasser and with me about how to deal with each other; In process; Bathroom renovation over a year after the fact; There were conflicting responses from the faculty; I have not yet decided whether or not I want to move forward

Examples of statements, separated by semicolon (right), the assigned category by research team (middle), and coded value for analysis (left).

Respondent status. Respondent status at the time of the incident was coded into 16 categories that ranged from graduate student to tenured faculty. These categories were based on the open-ended responses provided on the survey by the respondents (see Table 7 for categories and examples).

**Table 7:** Respondent status codes at time of reported harassment.

Coded Value	Assigned category	Example responses*
1	Assistant Professor	tenure track; visiting assistant professor; untenured; junior faculty; research assistant professor; pre-tenure
2	Associate Professor	tenured associate professor; just tenured; tenured faculty; tenured professor
3	Full Professor	full tenured professor; full professor; department chair; dean
4	Adjunct Professor	adjunct instructor; adjunct professor; adjunct lecturer; adjunct faculty; contingent faculty; temporary faculty
5	Lecturer	instructor; visting lecturer; teaching fellow; visiting scholar; visiting faculty; off-tenure track; non-tenure track; part-time professor; senior lecturer; teaching staff
6	Graduate Students (PhD)	ABD; PhD student; PhD candidate; doctoral student; doctoral candidate; prospective PhD student; 5th-year graduate student; MA/PhD student; TA/PhD student; visiting graduate student fellow; PhD admit
7	MA Student	Masters student; MFA student; MA student; MSc student; MBA student; pre-Masters student; MS student
8	Undergraduate	undergrad TA; undergraduate student; freshman in college; sophomore in college; junior in college; graduating senior; applying to graduate schools; 5th-year undergrad; post-bacc; undergraduate research assistant; 20-year-old student; BA student; college student
9	Staff	research technician; admnistrator; staff manager; employee; librarian; project manager; curator; registered nurse; teacher; director; coordinator
10	Postdoctorate	post-doctorate student; research postdoc; post-graduate; administrative postdoc;
11	High School	high school student; middle school student taking college classes
12	Multiple Status	PhD student/Assistant Professor; graduate student and undergraduate student; assistant and associate; graduate student through assistant professor; student and then post-grad; at all levels
13	Graduate Students	graduate student; research assistant; grad student; graduate school applicant; MA student applying to PhD programs; teaching assistant; post-MA; graduate teaching assistant; fresh out of grad school; law student; candidate for advanced degree; medical student
14	Faculty, Professor	professor; faculty; colleague/professor; scholar
15	Other	NA; witness; single; visitor; job candidate; job applicant; sober; trainee; naive; independent scholar; conference participant; research scientist; surgical resident; acquaintances of the students
16	Student	student; student victims; student workers

*Typos and misspellings left uncorrected. Examples of statements from original data, separated by semicolon (right), the assigned category by research team (middle), and coded value for analysis (left).

Perpetrator’s status. The reported perpetrator’s status was coded into 8 categories, based on the open-ended responses provided on the survey by the responders. Some perpetrators had multiple roles (ex: a professor AND a dean) or relationship with the responder (e.g., a professor AND an advisor, see Table 3.)

### Data analysis

#### Logistic regression model specification (RQ1)

We used respondent, perpetrator, and institutional characteristics to estimate the likelihood of subgroups’ choice to not report sexual harassment. We specified a logistic regression model (Eq 1, below) with perpetrator characteristics (β_1–9_), respondent characteristics (β_10–15_), and institution type (β_16_). Variables were simultaneously entered into a logistic regression model using Stata 15.

$$Log\left(\frac{Y}{1-Y}\right)=\beta_{0}+\beta_{1-9}PERPETRATOR\_CHAR+\beta_{10-15}RESPONDANT\_CHAR+\beta_{16}INSTITUION\_TYPE+\varepsilon$$ (1)

The primary coefficients of interest were β_10–15_, which represent disciplinary affiliations of respondents. Coefficients of secondary interest were β_1–9_, which represent perpetrator characteristics (e.g., tenure status). Institutional type was operationalized as a control variable (β_16_), since the original survey categories do not follow traditional Carnegie Classifications for institutions of higher education [49]. We are, moreover, unaware of extant literature that speaks to a pattern of institutional differences regarding sexual harassment reporting and would thus warrant a different approach.

#### Sentiment analysis (RQ2)

Our analysis used dictionary-based semantic annotations to assign a sentiment score ranging from -1 (negative) to 1 (positive) to each of the open-ended responses. This approach was the most appropriate, given that we do not know of a collection of text responses that we could use as a reference category to use more sophisticated methods [50]. Different sentiment analysis scores suggest different emotional states and intensity of language used. Descriptive heatmaps were generated by plotting sentiment scores along two dimensions: 1) institution type, and 2) demographic characteristics of respondents. Heatmaps in the results section were generated using R. (See Table 8 for summary statistics of sentiment scores.)

**Table 8:** Summary statistics of sentiment scores.

	N	Mean	SD	Min	Max
**Students**	1,596	0.05	0.12	0.60	1.00
General Narrative Sentiment	1,319	0.03	0.22	-1.00	1.00
Career Impact Sentiment	1,322	-0.12	0.35	-1.00	1.00
Mental Impact Sentiment	1,180	0.06	0.21	-1.00	1.00
Life Choices and Trajectory Sentiment					
**Faculty/Staff**					
General Narrative Sentiment	676	0.04	0.12	-0.64	0.43
Career Impact Sentiment	599	-0.01	0.25	-1.00	1.00
Mental Impact Sentiment	597	-0.16	0.35	-1.00	1.00
Life Choices and Trajectory Sentiment	528	0.03	0.22	-1.00	1.00
**Combined**					
General Narrative Sentiment	2,272	0.05	0.12	-0.64	1.00
Career Impact Sentiment	1,918	0.01	0.23	-1.00	1.00
Mental Impact Sentiment	1,919	-0.13	0.35	-1.00	1.00
Life Choices and Trajectory Sentiment	1,708	0.05	0.21	-1.00	1.00

## Results

### Institutional response

Institutional responses to respondents reporting sexual harassment included: some action taken (9%), no action taken (34%), retaliation (8%), and unclear if action taken (6.5%). Choosing to not report the incident at all was the most common response (36%). This decision varied across disciplines, with roughly 50% of respondents in the physical sciences and engineering choosing to not report the incident. In contrast, only 18% of staff chose to not report the incident (see Table 9).

**Table 9:** Reported institutional responses to harassment.

	None	Did not Report	Action Taken	Unclear	Retaliation	Other
Humanities	40.7%	35.3%	7.4%	6.9%	6.2%	3.5%
Social Sciences	40.4%	34.6%	10.0%	7.5%	4.2%	3.3%
Physical Sciences	37.1%	47.2%	7.0%	2.1%	5.2%	1.4%
Engineering	30.0%	50.0%	13.3%	6.7%	0.0%	0.0%
Professional	35.0%	35.8%	4.2%	11.7%	10.0%	3.3%
Staff	22.7%	18.2%	13.6%	4.5%	22.7%	18.2%
Average	34.3%	36.9%	9.3%	6.6%	8.1%	5.0%

**None** = no institutional response, unclear if reported; **Did not report** = respondents indicated incident was not reported; **Action Taken** = Reported, some action was taken by institution; **Unclear** = unclear if the incident was formally reported, unclear response to reporting; **Retaliation** = reported retaliation against respondent. **Other** = responses do not fall into one of the above categories.

### Logistic regression

Results from logistic regression analysis indicate that students were 1.6 times more likely to not report their experiences when compared with faculty and staff respondents (p < .001). Students in the physical sciences were 1.7 times more likely to not report their experiences when compared to students in other disciplines (p < .05). If the perpetrator was identified as a faculty member, then respondents were 1.5 times more likely to not report the incident compared to respondents who identified the perpetrator as a graduate student, postdoc, or other non-faculty (p < .001). Institution type (e.g., Elite/Ivy, Other R1) was not predictive of respondents choosing to not report sexual harassment (see Table 10 for full logistic regression results).

**Table 10:** Logistic regression results.

	β	SE	e^β^	95% CI of e^β^	
Constant	-1.10	.11	.38	0.21	0.68
**Role of Perpetrator**					
Advisor/Mentor	-.04	.17	.97	0.68	1.37
Chair/Head of Department	-.35	.14	.70	0.48	1.03
Tenured	.06	.13	1.06	0.83	1.35
Dean	.08	.57	1.09	0.39	3.03
Principal Investigator	-.30	.36	.74	0.29	1.92
Endowed/Named/Famous	-.11	.22	.89	0.56	1.43
Other Supervisory Role	-.28	.13	.76	0.54	1.06
Faculty^†^	.43**	.20	1.53**	1.18	1.99
Male	-.23	.16	.80	0.54	1.17
**Role of Respondent**					
Student	.39***	.18	1.47***	1.16	1.87
Humanities	.02	.22	1.02	0.67	1.54
Social Science	-.02	.22	.98	0.63	1.52
Physical Sciences	.54*	.40	1.72*	1.09	2.72
Engineering	.63	.81	1.88	0.81	4.39
Staff	-.39	.41	.67	0.21	2.19
**Institution Type**^**‡**^					
Other R1	-.04	.12	.96	0.75	1.22
Elite Institution/Ivy	.14	.16	1.15	0.88	1.50
N = 1,675					

Note

* p < .05

** p < .01

*** p < .001.

† = Any type of faculty; types coded as dichotomous.

‡ = Compared to reference group, consisting of an aggregate of: “Other Research Agency, Other Type of School, R2, Regional Teaching College, Small Liberal Arts College, [redacted], More than one institution”

### Descriptive sentiment analysis

**Fig 1:**
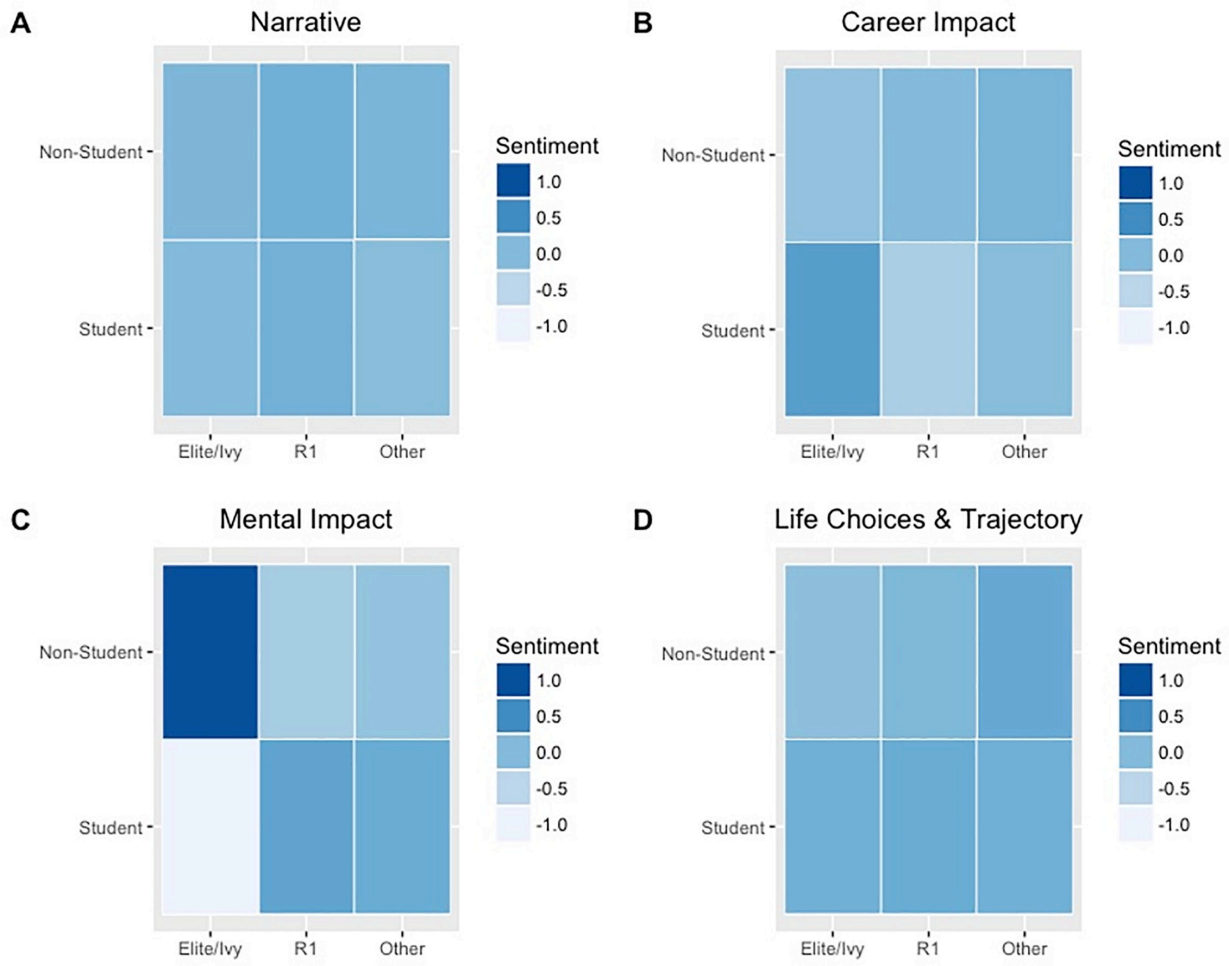
Sentiment score heatmaps of harassment narrative by student/non-student. (A), career narrative (B), mental health narrative (C), and life trajectory narrative (D), groped by student and non-student and institution type.

**Fig 2:**
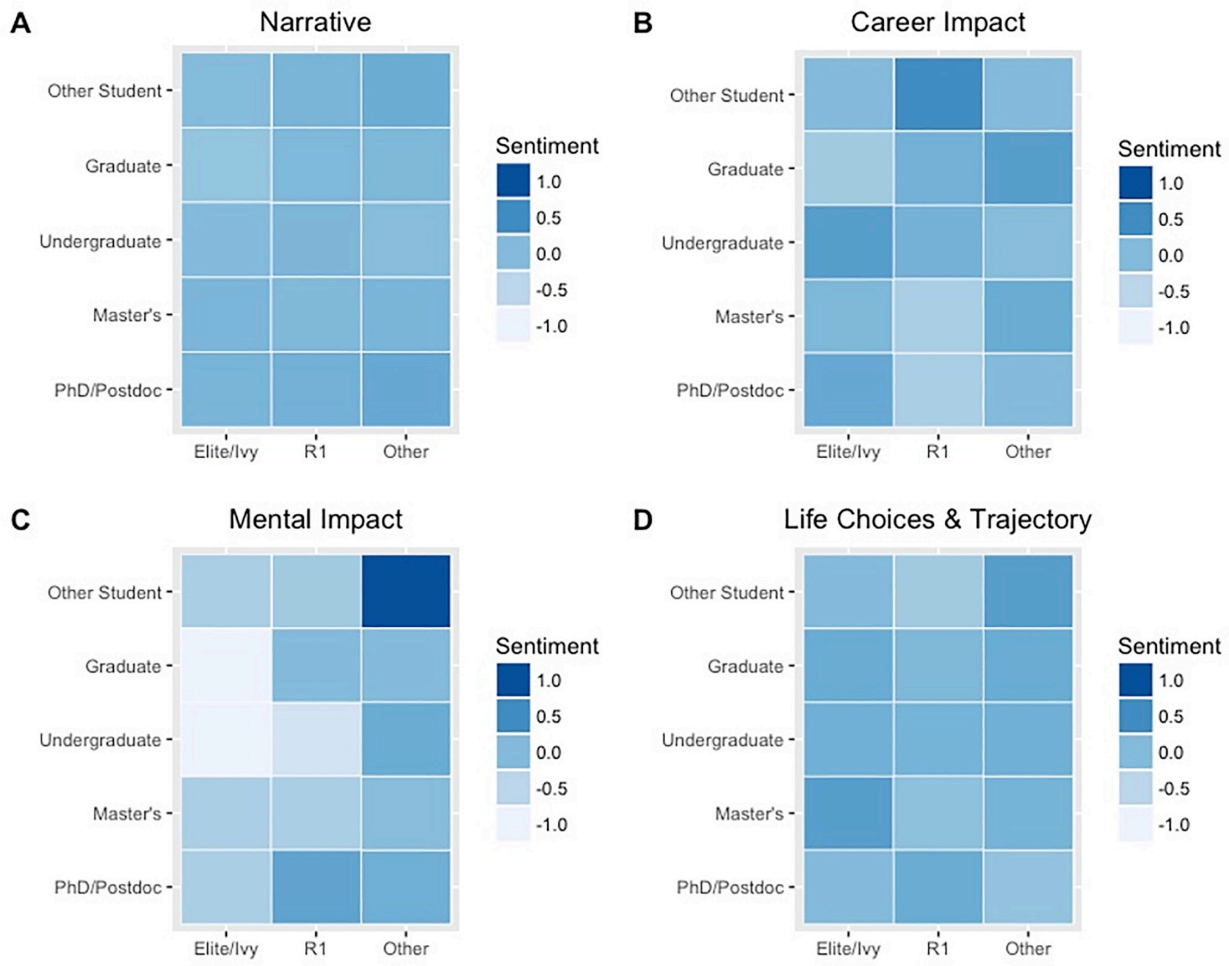
Sentiment score heatmaps of harassment narrative by student status. (A), career narrative (B), mental health narrative (C), and life trajectory narrative (D), grouped by student type and institution type.

**Fig 3:**
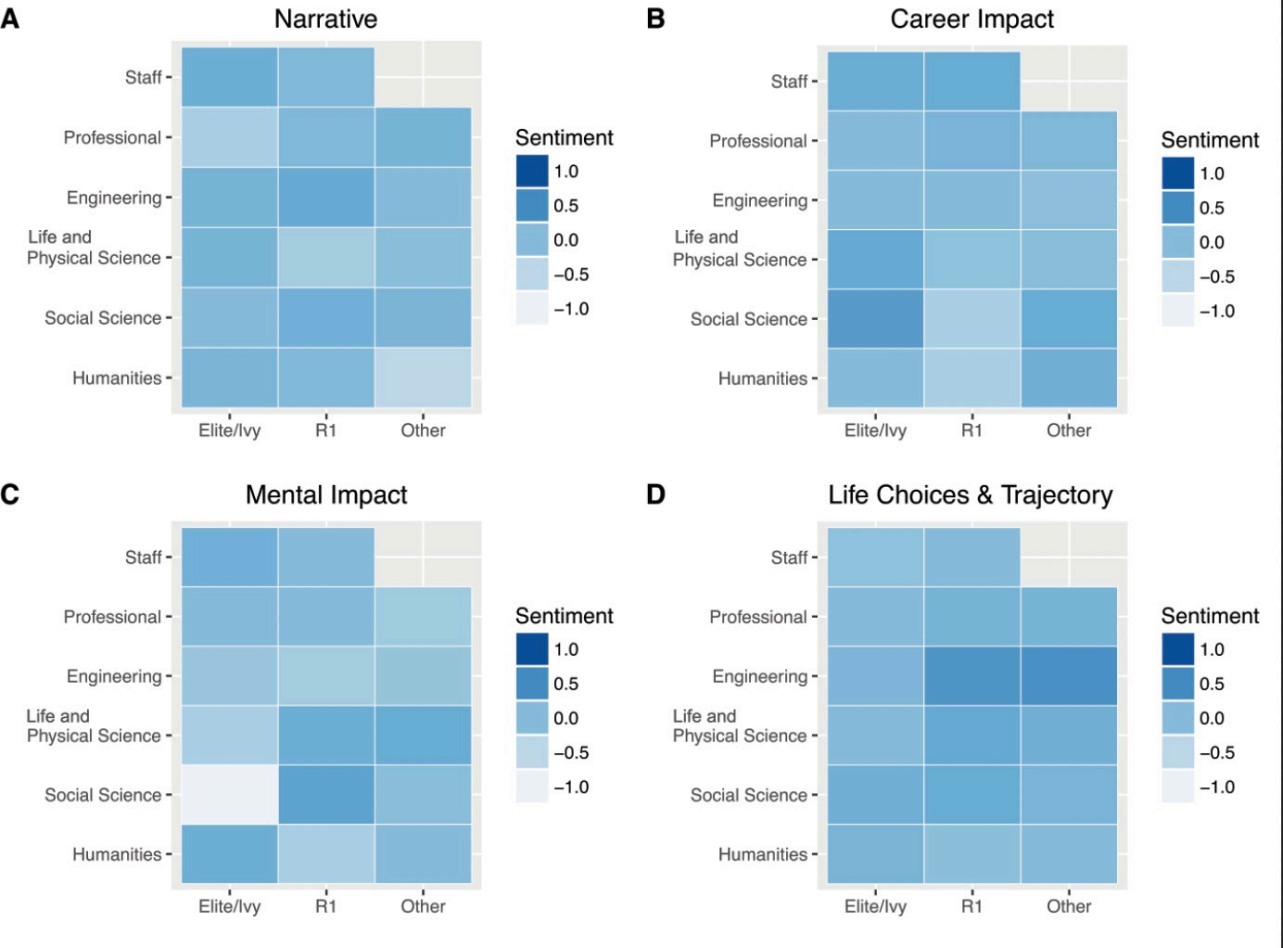
Sentiment score heatmaps of harassment narrative by discipline. (A), career narrative (B), mental health narrative (C), and life trajectory narrative (D), groped by student type and institution type.

Descriptive analysis of the open-ended responses (RQ2) indicated suggests variation in the mental health narratives, especially for students attending elite/Ivy League institutions as compared to other respondents (Fig 1C). A further breakdown analysis indicated that this pattern varied most among undergraduate and graduate students who attended elite/Ivy League institutions (Fig 2C). Sentiment scores of the mental health narrative were more negative for students in the social sciences who attended elite/Ivy institutions when compared to students attending other institution types of institutions, regardless of discipline (Fig 3C).

## Limitations

We note that the data that underpin our analysis were taken from a crowdsourced survey that was primarily distributed via social networks (e.g., Facebook), and as such likely suffers from selection bias. Specifically, it is likely that respondents to the survey have a tendency to use social networks regularly and to engage in social networks. Respondents were also likely familiar with Dr. Karen Kelsky’s service and/or somehow connected to one or more of her social network accounts. This suggests that, as a group, they may have a similar set of experiences [51]. This is born out in the data itself; nearly half of the respondents are from Humanities (Table 2) and other members of academia were underrepresented (e.g., administrative staff).

Some respondent characteristics (e.g., race, sexual orientation, gender, age at the time of incident) and key dates (e.g., the year of the incident), moreover, were not captured by the survey. This is unfortunate, since such information would have enabled us to examine how reported experiences differed as a function of individual’s demographic characteristics and/or policies that would have varied over time (e.g., the 1980’s vs. today). Respondents’ decision to report—and the reporting procedures available to them—have likely differed over time as a result of changes to EEO and Title IX policies and interpretations. In addition, retrospection bias (e.g., [52–53]), affects the reliability of the respondents’ accounts. As such, we note that we do not make strong claims regarding the intensity of respondents accounts; our logistic regression analysis, for example, is limited to a subset of reporting behaviors (i.e., not reporting). Our covariates were chosen similarly so that we could mitigate against the influence of any retrospection bias.

There are some challenges associated with the use of sentiment analysis as there are with all methods. One of the challenges pertains to the context of the text such that a particular word’s meaning might vary depending on the context (e.g., a word that is positive in one context might be negative in a different context), or a lack of context in texts [54]. It is also difficult to detect subtle and implicit social cues such as humor and sarcasm, as well as inconsistencies in written texts—such as contradictory statements [54]. We emphasize that our sentiment analysis is descriptive, as we did not use scores in subsequent inferential statistical analysis.

## Discussion

Our study supports to the notion that when sexual harassment occurs in the academy, it often does so via power asymmetries, i.e., scenarios which the perpetrator of sexual harassment is in a position of power relative to the person who is sexually harassed. Specifically, student respondents were more likely to avoid reporting sexual harassment. Similarly, despite being underrepresented in our sample, respondents who identified as staff reported more retaliation (Table 9). Previous work has shown that power imbalances can elicit situations associated with high rates of harassment and underreporting [1, 10, 55]. Newins & White [16], for example, found that over a third of students (36%) were unsure whether they would tell a faculty member about sexual assault, and about a fifth (16% to 22% percent) were not willing to disclose at all.

The variability of underreporting behaviors across disciplines also suggests differences in disciplinary culture—the fact that only 50% of respondents in STEM fields chose to report their incident suggests that there are potential barriers to doing so, and may also suggest a process that makes reporting undesirable, a point underscored by the #MeTooSTEM movement and previous work [22, 56]. Representation likely plays a role, as decades of work shows that women are underrepresented in STEM (e.g., [57–60]). Low representation of women, in addition to higher rates of experiencing sexual harassment, may thus create a difficult environment for women in STEM.

Sentiment analysis of written accounts helps to uncover additional information within a data set ([36–37]), and our descriptive analysis suggests institutional differences among the mental health narratives (Figs 1–3). These differences were most apparent when examining sentiment scores of students who attended social science programs within elite/Ivy League institutions (Fig 3). We infer from this analysis that one must also attend to institutional differences when investigating sexual harassment and its impact on the academic community. Such studies should attend to differences in implementing and reinforcing policies regarding sexual harassment [48, 61–63]. However, our study cannot definitely state that the apparent institutional differences in respondents’ sentiments regarding the mental impact of sexual harassment were due to true institutional differences or were in fact an artifact of how data were collected. Regardless, it is imperative for each institution to provide pathways that can best support those impacted by sexual harassment and provide necessary services, such as counseling. Compulsory reporting practices, if in place, should also be examined so that the well-being of those who report is protected.

It is important to address sexual harassment in the academy; those who choose to sexually harass their students and/or colleagues are responsible for a host of negative outcomes among those they victimize. Chronic sexual harassment has been shown to be predictive of anxiety, depression, and substance abuse; negative health outcomes include hypertension and poor sleep [64–65]. Sexual harassment is also associated with psychological duress and lower academic satisfaction [66]. Men are not immune from sexual harassment in academia, but experience it at lower rates compared to women [4].

Recent work has highlighted the fact that women feel more supported and/or empowered to speak of when they experience sexual harassment [67]. Notably, one of the themes that emerged in the Keplinger et al. [67] indicates that the #MeToo movement has helped many feel comfortable with sharing sexual harassment experiences. Troublingly, however, while sexual harassment has decreased in the wake of the #MeToo movement, gender harassment (defined as negative views of women and gender hostility) has increased [67]. Universities should thus continue to pay close attention to gender harassment to ensure that it does not increase as sexual harassment decreases. Future work in this area might also examine differences between the reported sexual harassment experiences of women before and after the #MeToo movement. It is also crucial for future work to examine the reported sexual harassment experiences of populations may be particularly vulnerable to sexual harassment, such as LGBTQ community, people with disabilities, and people of color. Such work could reveal whether the institutions have made effective and tangible changes to the ways they handle sexual harassment. Taking such steps will help to ensure that the next generation of scientists are trained in an environment where safety and respect are the norm.
